# Depolarization-Associated CircRNA Regulate Neural Gene Expression and in Some Cases May Function as Templates for Translation

**DOI:** 10.3390/cells9010025

**Published:** 2019-12-20

**Authors:** Ebrahim Mahmoudi, Dylan Kiltschewskij, Chantel Fitzsimmons, Murray J. Cairns

**Affiliations:** 1 School of Biomedical Sciences and Pharmacy, University of Newcastle, Callaghan, NSW 2308, Australia; ebrahim.mahmoudi@uon.edu.au (E.M.); Dylan.Kiltschewskij@uon.edu.au (D.K.); Chantel.Fitzsimmons@newcastle.edu.au (C.F.); 2 Centre for Brain and Mental Health Research, University of Newcastle, Callaghan, NSW 2308, Australia; 3 Hunter Medical Research Institute (HMRI), New Lambton, NSW 2305, Australia

**Keywords:** circular RNAs (circRNAs), circ-EXOC6B, depolarization, expression, translation

## Abstract

Circular RNAs (circRNAs) are a relatively new class of RNA transcript with high abundance in the mammalian brain. Here, we show that circRNAs expression in differentiated neuroblastoma cells were significantly altered after depolarization with 107 upregulated and 47 downregulated circRNAs. This coincided with a global alteration in the expression of microRNA (miRNA) (*n* = 269) and mRNA (*n* = 1511) in depolarized cells, suggesting a regulatory axis of circRNA–miRNA–mRNA is involved in the cellular response to neural activity. In support of this, our in silico analysis revealed that the circular transcripts had the capacity to influence mRNA expression through interaction with common miRNAs. Loss-of-function of a highly expressed circRNA, circ-EXOC6B, resulted in altered expression of numerous mRNAs enriched in processes related to the EXOC6B function, suggesting that circRNAs may specifically regulate the genes acting in relation to their host genes. We also found that a subset of circRNAs, particularly in depolarized cells, were associated with ribosomes, suggesting they may be translated into protein. Overall, these data support a role for circRNAs in the modification of gene regulation associated with neuronal activity.

## 1. Introduction

Circular RNAs (circRNAs) are covalently closed-loop RNA molecules that are formed by back-splicing of the 5′ and the 3′ ends of a primary transcript [1]. Development of high-throughput sequencing technology has helped systematic identification of these species in mammals, including humans and mice, as well as other eukaryotic clades, such as insects and plants [2,3,4]. CircRNAs arise from exonic and/or intronic regions and span between 100 to 4000 bp in length [5]. While these molecules are distributed throughout the genome, the majority of circRNAs are produced from coding segments [5]. Emerging evidence suggests that circRNAs are functionally significant for the cell and display high levels of localization at both the tissue level and in respect to different subcellular compartments, including cytoplasm and nucleus [5]. It has been suggested that these transcript isoforms are important for supporting hemostasis as well as asserting a key regulatory role in cellular processes, such as embryonic development, cell signaling, control of cell cycle, and response to cellular stress [6]. Perturbations of their expression are also being reported in association with many diseases including cancer and neurobiological disorders such as schizophrenia [7,8,9,10]. CircRNA have been implicated in several regulatory mechanisms from transcription through to translation but seem to be a particularly active substrate for absorbing small non-coding miRNA and thus represent a significant matrix of competing endogenous RNA (ceRNA) [5]. These molecules are highly stable and capable of encoding multiple antisense sequences that sponge a plethora of small RNAs and prevent them from interacting with their mRNA targets. CircRNA CDR1, for example, was confirmed to contain over 70 conserved target sites for the miR-7, a brain enriched miRNA, and to regulate numerous miR-7 target genes [11,12,13] through sponging the miRNA and reducing its intracellular bioavailability [1,14]. Interestingly, CDR1-dependent repression of mir-7 can be rescued by another miRNA, miR-671, which is thought to be capable of Argonaute (AGO)-mediated cleavage of CDR1as, resulting in release of its miR-7 cargo [14]. Collectively, circRNA can bind a large proportion of miRNA species and are thought to provide the basis of a very substantial ceRNA network modulating miRNA function. In addition to miRNA sponging, circRNA have been shown to regulate the function of RNA-binding proteins [15] and also can modify the transcription rate of their own host gene [16]. It was recently suggested that circRNAs are not exclusively noncoding RNA; they can serve as templates for the translation of bio-active polypeptides [17,18,19]. The presence of open reading frames (ORFs) as well as internal ribosome entry sites (IRESs) within endogenous circRNAs enables these molecules to produce functional peptides through a cap-independent mechanism [20,21]. However, the scope of circRNA translation is not yet clear.

CircRNAs are expressed in various cell lines and tissues, particularly in the nervous system, where they reach their greatest number and concentration [2,22,23]. These molecules are dynamically regulated during neurodevelopment and are thought to be active in neural differentiation and synaptic plasticity [2,24]. In the current study, we investigated the expression and the regulatory role of circRNA in the response to potassium-induced depolarization in differentiated human neuroblast cells. We observed that circRNAs are differentially expressed in response to depolarizing stimulus that is associated with significant changes in miRNA and mRNA expression. Interactions between these circRNAs and their cognate miRNA (and their relationship to target mRNA) suggested that these neural activity-associated circRNAs form an influential ceRNA regulatory axis related to neural function. Through loss-of-function analysis, we show that these circRNAs can specifically regulate gene expression, possibly through sponging miRNA. We also found that a subset of circRNAs in depolarized cells associate with ribosomes and may act as templates for translation.

## 2. Materials and Methods

### 2.1. Cell Culture and Differentiation

SH-SY5Y human neuroblastoma cells were cultured at 37 °C, 5% CO2, 90% humidity in Dulbecco’s Modified Eagle’s Medium (DMEM, Hyclone) supplemented with 10% fetal calf serum (FCS, Sigma-Aldrich, St. Louis, Missouri, MO, USA), 1% L-glutamine, and 2% HEPES. Cells were harvested by washing with phosphate buffered saline (PBS) followed by incubation with trypsin for one minute. To obtain neuronal cells, populations were differentiated as follows. First, 24 h after seeding cells into new culture flasks, new medium supplemented with 10 µM all-trans retinoic acid (ATRA, Sigma) was added, and cultures were subsequently incubated and wrapped in foil for 5 days while media was changed on day 3. After 5 days, ATRA was removed by washing 3 times with DMEM. The neuronal differentiation was confirmed by the high expression of neural marker genes by mRNA sequencing; the average numbers of reads were as follows: TUBB3 (36,121); ENO2 (13,922); MAP2 (6946); MAPT (2033) and SV2A (6410).

### 2.2. Depolarization

Depolarization was induced by 3 min room temperature incubation in stimulating HEPES buffered saline (HBS) (35 mM NaCl, 100 mM KCl, 0.6 mM MgSO4.7H2O, 2.5 mM CaCl2.2H2O, 10 mM HEPES, 6 mM glucose) [25]. The HBS was then replaced with warm complete medium, and cells were incubated for 10 min under culturing conditions to recover. Additionally, sham-depolarized controls (resting condition) were provided using a non-stimulating HBS from which KCl was removed and NaCl was increased to 140 mM. We used 4 biological replicates per group (depolarized and resting) for mRNA and miRNA and 3 biological replicates per group for circRNA experiments.

### 2.3. RNA Extraction and Quality Control

Total RNA from depolarized and resting differentiated cells was extracted using TRIzol (Invitrogen) with an enhanced −30 °C precipitation in isopropanol for at least 2 h using glycogen (Sigma) as a co-precipitant. RNA quality was analyzed using an Agilent Bioanalyzer RNA 6000 Nano chip, and small RNA quality was checked by Bioanalyzer Small RNA chip before sequencing library preparation.

### 2.4. RNA-Seq Library Generation and Sequencing

For circRNAs enriched sequencing, 5 μg of total RNA [RNA integrity number (RIN) ≥ 8.5] was treated with DNase I (1U/μg RNA, Thermo Scientific, Waltham, MA, USA) and then depleted for ribosomal RNA using the Ribo-Zero kit (Illumina, San Diego, CA, USA). Next, linear RNAs were degraded via RNase R (3U/μg, Epicenter, Madison, WI, USA)performed at 37 °C for 15 min. Sequencing libraries were then constructed using the Illumina TruSeq Stranded Total RNA Library Prep Kit according to the manufacturer’s protocol, after which library quality and concentrations were determined using an Agilent High Sensitivity DNA Bioanalyzer chip. Libraries were all pooled and run on NexSeq500 instrument with 150 cycles of single-end reads. For mRNA sequencing, 1 μg DNase treated total RNA (RIN ≥ 8.5) was subjected to mRNA isolation using oligo-dT beads (Illumina Heat fragmentation, 94 °C, 8 min). Library preparation was conducted using the TruSeq Stranded mRNA Library Prep kit (Illumina), and quality control of libraries was performed using Agilent 2100 Bioanalyzer before 150 cycles of single end sequencing on the NexSeq500 sequencer.

MiRNA sequencing was conducted using 1 μg of high quality (RIN ≥ 8.5) total RNA using the Illumina TruSeq Small RNA Library Preparation Kit. Briefly, adapter ligation, reverse transcription, and 11 PCR cycles were performed according to the manufacturer’s instructions. The successful production of small RNA amplicons was then confirmed via high sensitivity DNA chip (Agilent), after which all PCR products were run on a 6% native polyacrylamide gel, ~145–160 bp bands corresponding to miRNA were excised and shredded, and cDNA was eluted overnight in molecular grade water with gentle agitation. The following day, cDNA was extracted and quantified via high sensitivity DNA chip. Samples were subsequently pooled in equimolar ratios, denatured, and subjected to 75 cycles of single-end sequencing using the NextSeq 500 sequencing platform (Illumina). All the RNA sequencing experiments were performed in one technical replicate. The data are available for download from the gene expression omnibus (GEO) with accession number GSE139443.

### 2.5. Prediction and Quantification of CircRNAs

Raw sequencing data were demultiplexed based on unique adapter sequences and converted to fastq format using the “bcl2fastq” package (version 2.20, Illumina (https://support.illumina.com/sequencing/sequencing_software/bcl2fastq-conversion-software.html), ensuring adapter sequences were not masked. Sequencing read quality was then assessed using the FastQC sequencing quality control package (version 0.11.5, (https://www.bioinformatics.babraham.ac.uk/projects/fastqc), after which “cutadapt” (version 1.14) [26] was used to trim adapter sequences and remove low quality 3’ nucleotides. We used CIRCexplorer [27] to predict back-splice junction candidates. Briefly, sequence reads were aligned to human reference genome hg19 using TopHat (v2.0.9) [28] and then unmapped reads were extracted and aligned onto the reference genome using TopHat-Fusion [29] to collect the reads mapped on the same chromosome but in a reverse order, which were then considered as candidate back-spliced junctions. Finally, all the back-splice candidates were checked for supporting reads, and those with more than one read were regarded as circRNAs. Estimation of circRNAs expression was performed by quantifying the number of reads spanning back-spliced junctions (circular RNA reads). Next, we normalized the back-spliced junction read counts by sequencing depth in each sample. CircRNA reads were divided by the total number of mapped reads in each sample to obtain RPM (mapped back-splice junction reads per million mapped reads) values [30]. The relative expression of circRNAs was calculated by comparing RPM values between samples. One sample from the depolarized group was excluded from analysis after quality control (QC) due to a significantly lower number of the circRNAs as well as the lower abundance of back-spliced events (thus considered an outlier), leaving 2 replicates for this group.

### 2.6. mRNA and miRNA Expression Analysis

For mRNA expression analysis, raw reads from individual libraries were demultiplexed, quality checked and trimmed, as mentioned above, and then aligned to the human reference genome hg38 using TopHat (v2.0.9) Reads aligning to features were subsequently counted using htseq-count (v0.7.2) [31], and differential expression analysis was performed using the Bioconductor package EdgeR [32] for differential expression analysis. We also obtained the transcripts per million (TPM) values from normalized read counts in EdgeR through rpkm () function. For miRNA expression analysis, reads were mapped to the reference genome using the Bowtie2 (v2.2.6) short read aligner. Using the miRBase mature miRNA reference annotation, reads mapping to mature miRNAs were then counted using htseq-count, and differential expression was quantified using EdgeR.

### 2.7. cDNA Synthesis and Quantitative PCR

Total RNA treated with DNAse I (1 U/1 μg RNA, Thermo Scientific) was subjected to reverse transcription with Superscript II Reverse Transcriptase (Invitrogen, Carlsbad, CA, USA) using random hexamers according to the manufacturer’s instructions. To test the resistance of circRNAs to RNaseR, qRT-PCR was performed on RNA samples with and without RNaseR treatment. Using divergent primers specifically designed to detect back-splice junctions, RT-qPCR reactions were performed on an Applied Biosystems 7500 real-time PCR machine using Power SYBR green master mix (Thermo Fisher Scientific, Waltham, MA, USA) following the manufacturer’s instructions. GAPDH served as internal reference to normalize the data using the ΔCt method. All reactions were performed in triplicate and are presented as mean ± SEM. The Student’s t-test (one-tailed) was applied to compare expression levels. All the primer sequences for RT- PCR and qPCR are listed in Table S6.

### 2.8. Functional Enrichment Analysis

Gene ontology enrichment analysis was carried out for the host genes of the circRNAs and the differentially expressed (DE) mRNAs using GO (http://geneontology.org) [33]. We uploaded the host genes and the DE genes as input list and the *Homo sapiens* genes (*n* = 20996) as reference list for the analysis. The Fisher’s exact test was used for the statistical test, and false discovery rate (FDR) was applied for multiple test correction. A *p* -value < 0.05 and FDR < 0.05 was set to detect the significant clusters. Pathway enrichment analysis was additionally run using Reactome database version 65 [34]. A *p*-value < 0.05 and FDR < 0.05 was set to identify significant pathways.

### 2.9. MiRNA Binding Site Prediction and circRNA–miRNA–mRNA Network Construction

To predict miRNA binding sites within circRNAs, exon sequences of the circRNAs were extracted using hg19 annotation. Then, miRNA binding sites were identified using TargetScan_60 and targetscan_61_context_scores Perl scripts (http://www.targetscan.org/vert_71/) [35,36]. We used a context score threshold cutoff < −0.2 to obtain reliable interactions. We made the circRNA–miRNA interaction pairs for the DE circRNAs and the DE miRNA by predicting the miRNA targets, as mentioned earlier. We considered the circRNA–miRNA pairs regardless of the expression patterns between circRNAs and miRNAs with the assumption that the expression of circRNAs may not affect the expression of miRNAs in sponging condition. Then, we generated the miRNA–mRNA interaction pairs applying the same strategy; the DE mRNA and the DE miRNA were included, and the miRNA binding targets were predicted according to the above criteria. We only considered the pairs with inverse expression directions. The circRNAs–miRNA–mRNA network was generated by combining the circRNA–miRNA pairs and the miRNA–mRNA pairs such that the common miRNAs served as linker between the DE circRNAs and the DE mRNAs. For subnetwork analysis, we ranked the nodes by their network feature using 11 topological analysis methods and then selected the top nodes to make the network. We used CytoHubba plugin [37] in Cytoscape tool for this analysis. All the interaction networks were visualized using Cytoscape tool v.3.5.1 (http://www.cytoscape.org/) [38].

### 2.10. circRNA Knockdown

Silencing of circ-EXOC6B (exocyst complex component 6B) and circ-DROSHA (drosha ribonuclease III) was performed using siRNAs synthesized by GenePharma. The sequences of the siRNAs were 5’-AUCCGUCACUUGAUUUUGCUU-3’ and 5’-UCGCGCAGGUCAGUUAAGUUU-3’ for circ-EXOC6B and circ-DROSHA, respectively. The designed siRNA oligos spanned the back-splice sequence to specifically target the circular transcripts of interest. After the design, we blasted the siRNA oligos and ensured that they only hit the expected targets. SH-SY5Y cells were differentiated as described and transfected by Cell Line Nucleofector Kit (Lonza, Basel, Switzerland) according to the manufacturer’s instruction. Briefly, 1.5 × 10^6^ cells were resuspended in 100 μL of nucleofector solution and co-transfected with 30 μM of siRNA and 2 μg pmaxGFP plasmid (Lonza). For the control samples, a scramble siRNA and a GFP plasmid were co-transfected. The G-004 program was used on the Nucleofector II device (Amaxa, Cologne, Germany). Immediately after nucleofection, cells were plated onto six-well plates and incubated under culturing conditions. The knockdown efficiency was tested using specific divergent primers after 24 h, and an 80% reduction in the abundance of circ-EXOC6B as well as a 60% for circ-DROSHA were observed as compared to the scramble siRNAs used as controls. The specificity of the siRNAs was further confirmed by checking the expression of potential off-targets (defined as all the blast hits, including those with the lowest possibility of interaction with the siRNAs), including DPY19L3, TMEM87A, MARCH1, SHC3, and MBNL3, and we found no change in expression in these genes in the RNAseq data. Also, there were no changes in the expression of the linear transcripts of the genes of interest (EXOC6B and DROSHA), ensuring they were not targeted by the siRNAs.

### 2.11. Ribosome Profiling

Ribosome profiling was performed using the Illumina TruSeq Ribo Profile kit (H/M/R) according to Ingolia et al. [39]. The differentiated SH-SY5Y cells (15 samples for depolarized and four for resting condition) were treated with cycloheximide-supplemented culture medium (0.1 mg/mL) for 1 min and subsequently washed with cycloheximide-supplemented ice-cold PBS (0.1 mg/mL). Cell lysates were extracted using Mammalian Lysis Buffer (1x Mammalian Polysome Buffer, 1% Triton X-100, 1 mM DTT, 1 U/µL DNase I, 0.1 mg/mL cycloheximide, 0.1% NP-40) and subsequently triturated (22-gauge needle) and clarified by centrifugation (17,000× *g*, 4 °C, 10 min). The unprotected RNA was degraded by TruSeq Ribo Profile Nuclease (RNase I; 90 units, 45 min at 23 °C) followed by adding 15 µL SUPERase-In RNase Inhibitor (Life Technologies, Carlsbad, CA, USA) to inhibit RNase activity. The protected RNA fragments in addition to small RNAs were subsequently fractionated by size exclusion chromatography using Illustra MicroSpin S-400 columns (GE Healthcare, Pittsburge, PA, USA). The resultant RNA was purified using RNA Clean and Concentrator-25 kit (Zymo Research) according to the manufacturer’s instructions, with minor amendments made by Illumina. After ribosomal RNAs removal, the 27–33 nt RNA fragments were cut, eluted from the gel, and used to construct sequencing libraries. The sequencing was run on NexSeq500 instrument (Illumina) with 75 cycles of single-end reads.

### 2.12. Analysis of Ribosome Profiling Data

Data quality was assessed using the FastQC, after which cutadapt (http://journal.embnet.org/index.php/embnetjournal/article/view/200) was used to trim adapter sequences, retain reads between 25 nt and 40 nt, remove low quality 3’ nucleotides, and trim singular 5’ nucleotides. Over-represented sequences derived from rRNA, miRNA, snoRNA, and snRNA were discarded, and the remaining reads were used to identify circRNAs. The reads were first aligned to the reference genome hg19 with Tophat 2.0.10 (tophat2 -a 6 --microexon-search –m 2), and unmapped reads were extracted. Theses reads were then aligned to an index of back-splice junctions (over 80,000 unique back-splice sites of 36 nt in length) created from our previous datasets using Bowtie 1.0.0 (parameters: bowtie -y --chunkmbs 128 -a --best --strata), and all the mapped reads were collected and considered as circRNAs.

### 2.13. Annotation of ribo-circRNAs and Prediction of Coding Sequence and Domains

To annotate the ribo-circRNAs we used the genomic features including 5′-UTR, 3′-UTR, and CDS coordinates downloaded from UCSC table browser [40]. The ribo-circRNAs coordinates were then intersected to these features using the bedtools intersect function [41]. All the circRNAs that overlapped with any of the features were reported. To predict the coding sequence and the domains within the ribo-circRNAs, the exonic sequence of each circRNA was retrieved, and then each circRNA sequence was multiplied four times to allow rolling circle translation. To predict circRNA with potential protein coding, we used the coding potential calculator (CPC2) tool [42] and selected those circRNAs with “coding” label. Predictions of conserved domains within the coding regions of ribo-circRNAs were performed using the Conserved Domain Database (CDD) [43].

## 3. Results

### 3.1. Annotation of circRNAs in Differentiated Neuroblastoma

**Figure 1:**
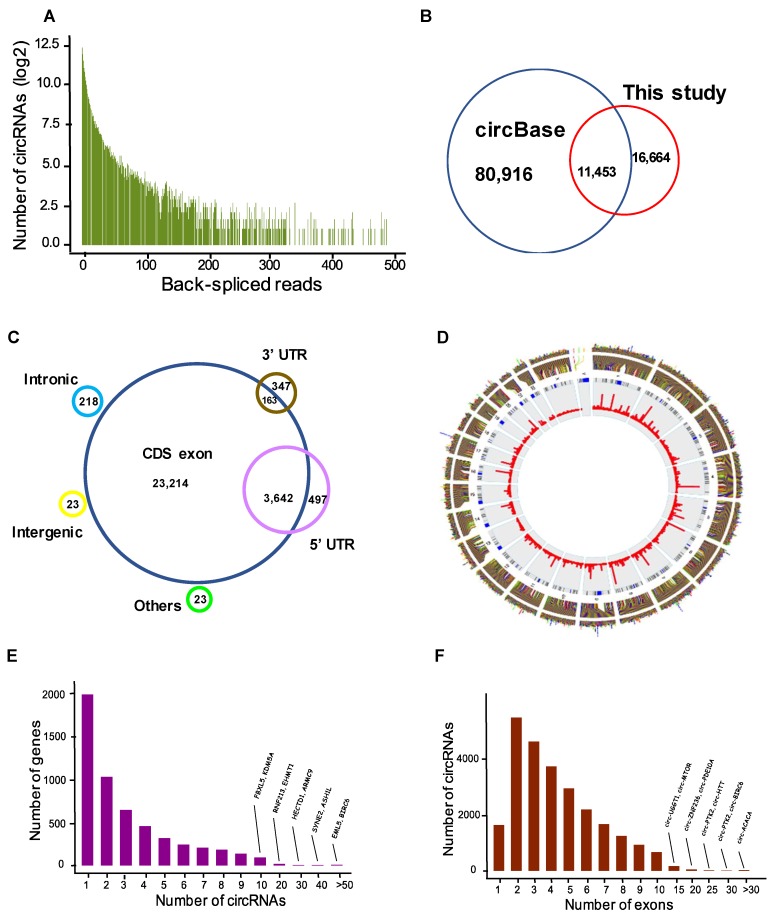
Annotation of the neuroblastoma circular RNAs (circRNAs). (**A**) The number of circRNAs and back-spliced reads identified in five samples of differentiated neuroblastoma. (**B**) Comparison of the identified circRNAs with the circRNA database circBase. (**C**) Genomic origin of the identified circRNAs. (**D**) Distribution and expression level of circRNAs per chromosome. The outer circle is circRNA host gene, the middle circle represents chromosome name, and the inner circle indicates bar graph of expression level of individual circRNAs. (**E**) Number of circRNAs produced per gene. (**F**) Histogram of the number of exons in the exonic circRNAs.

To determine genome-wide profiles of circRNAs in differentiated human SH-SY5Y neuroblast cultures depolarized with KCl, we performed RNA-sequencing using total RNA depleted of linear RNA using RNase R treatment. A total of over 300 million reads were generated for all samples with an average of 62 million per sample, with mappable reads ranging from 82.8 to 91.2%. CircRNA candidates were detected using the CIRCexplorer method, and to improve the confidence, we removed reads with fewer than two back-spliced junctions in at least one sample. In total, we identified 28,117 unique circRNA species ranging from two to 500 reads (Figure 1A and Table S1). After comparing these to the circRNA database circBase [44], comprising over 92,000 human circRNAs, we discovered 11,453 were already known. Surprisingly, an additional 16,664 circRNAs were novel with no previous annotation in circBase (Figure 1B and Table S1). Analysis of genomic features showed that, while circRNAs emerge from a variety of genomic segments, including intronic and untranslated regions, the vast majority (~90%) originate from protein coding exons (Figure 1C). Although circRNAs host genes are distributed across with the genome, they reach their greatest abundance in chromosomes 1, 2, 3, and 7 (not corrected for chromosome length). By contrast, chromosomes X, Y, 21, and 22 had the lowest number of circRNAs and lowest level of circRNAs expression (Figure 1D). While approximately 45% (1987) of parental genes produced only one circRNA, the remainder could yield two or more circularized transcripts including 11% with over ten distinct circRNAs, such as BIRC6 and ASH1L, which generated 97 and 40 distinct molecules, respectively (Figure 1E). Analysis of the relationship between the number of circRNAs per gene and the circRNA expression level (average) showed a positive relationship (Spearman’s rank correlation test: *p* < 2.2 × 10^−16^; Figure S1). The length of circRNAs varied from one exon to over 30 exons; however, 2–5 exon isoforms were dominant, accounting for 61%, and a lower fraction (10%) contained over 10 exons, such as circ-PTK2 and circ-ACACA (Figure 1F). We verified the authenticity of identified circRNAs by quantifying their resistance to RNase R using qPCR with specific divergent primers. All tested 10 circRNAs that were selected from very lowly to highly expressed circRNAs were found to be over 20-fold more resistant compared to the linear transcripts following the RNase R treatment (Figure S2).

### 3.2. CircRNAs are Differentially Expressed in Neuronal Depolarization

**Figure 2:**
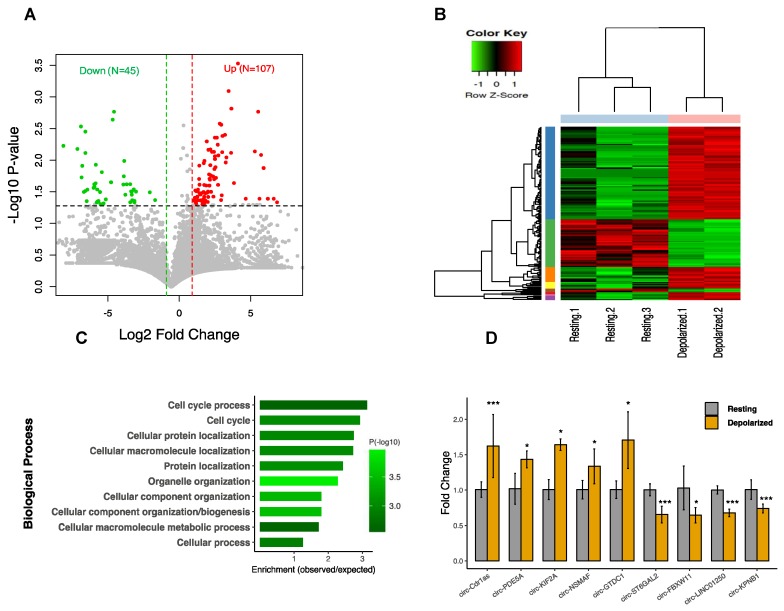
Differential expression of circRNAs in neuronal depolarization. (**A**) Volcano plot constructed using fold change values and *p*-values to compare the circRNA expression changes between depolarized and resting cells. Red dots represent significantly upregulated circRNAs, and green dots represent downregulated circRNAs, with the vertical lines corresponding to two-fold change and the horizontal line representing a *p*-value < 0.05 cut off. (**B**) Hierarchical clustering analysis of circRNA expression between depolarized and resting cells. Depolarized group contains two and resting group contains three samples. Expression values are represented in different colors where red shows upregulation and green shows downregulation. (**C**) Functional enrichment analysis of the differentially expressed (DE) circRNAs host genes. The top 10 significantly enriched biological process terms by gene ontology (GO) analysis. (**D**) Validation of circRNA differential expression for nine circRNAs using q-PCR. Data are shown as mean ± SEM. Reactions were performed in triplicate for each biological treatment group, and circRNAs expression was normalized using GAPDH as control. Statistical significance was assessed by Student’s t-test (one-tailed). *p < 0.05; *** *p* < 0.001.

Analysis of circRNA expression revealed a significant alteration in response to depolarization involving 152 differentially expressed (DE) circRNAs (*p*-value < 0.05 and fold change ≥ 2), including 107 upregulated and 45 downregulated (Figure 2A). The details of the altered circRNAs are provided in Table S2. Hierarchical clustering showed that the circRNA expression pattern was clearly distinguishable between resting and the depolarized conditions (Figure 2B). Gene ontology (GO) analysis of host genes from differentially expressed circRNA revealed enrichment of cellular process, such as cellular macromolecule metabolic process, cellular component organization/biogenesis, and cell cycle process (Figure 2C). To verify the circRNAs differential expression, we randomly selected eight altered circRNAs and performed qPCR with specific divergent primers. In addition, we tested the expression of CDR1as, a brain enriched circRNA with a key regulatory role in neuronal function [14]. The results showed a significant change of all the tested circRNAs in depolarized cells as compared to resting cells (Figure 2D).

### 3.3. CircRNA Regulation Coincides with Global Alteration of miRNA and mRNA

**Figure 3:**
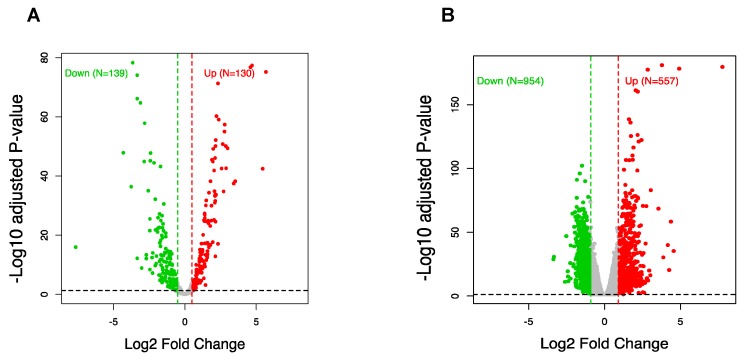
Differential expression of miRNA and mRNA in neuronal depolarization. (**A**) Volcano plot generated using fold change values and adjusted *p*-values [false discovery rate (FDR)] to compare the expression changes of miRNA and (**B**) mRNA between depolarized and resting cells. Red dots represent significant upregulation and green dots represent downregulation, with the vertical lines corresponding to 1.5-fold (miRNA) and two-fold (mRNA) change and the horizontal line representing an FDR cut off < 0.05.

CircRNA transcripts are known for their capacity to fine-tune miRNA function through their activity as ceRNA [45]. To observe this influence, we analyzed the expression profiles of miRNA and mRNA in response to depolarization using RNA sequencing. After mapping and aligning read counts, we further identified 269 miRNAs with differential expression after depolarization (FDR < 0.05, FC > 1.5) compared to the resting state cells. This consisted of 130 upregulated and 139 downregulated miRNAs (Figure 3A and Table S3). Also, we observed 1511 mRNA transcripts to be differentially expressed (FDR < 0.05, FC > 2), including 557 displaying upregulation and 954 with downregulation (Figure 3B and Table S3). When considered in respect to the observed change in circRNA expression, this suggested there is a regulatory relationship between circRNA, miRNA, and mRNA in the molecular response to depolarization.

### 3.4. CircRNA–miRNA–mRNA Association Network

**Figure 4:**
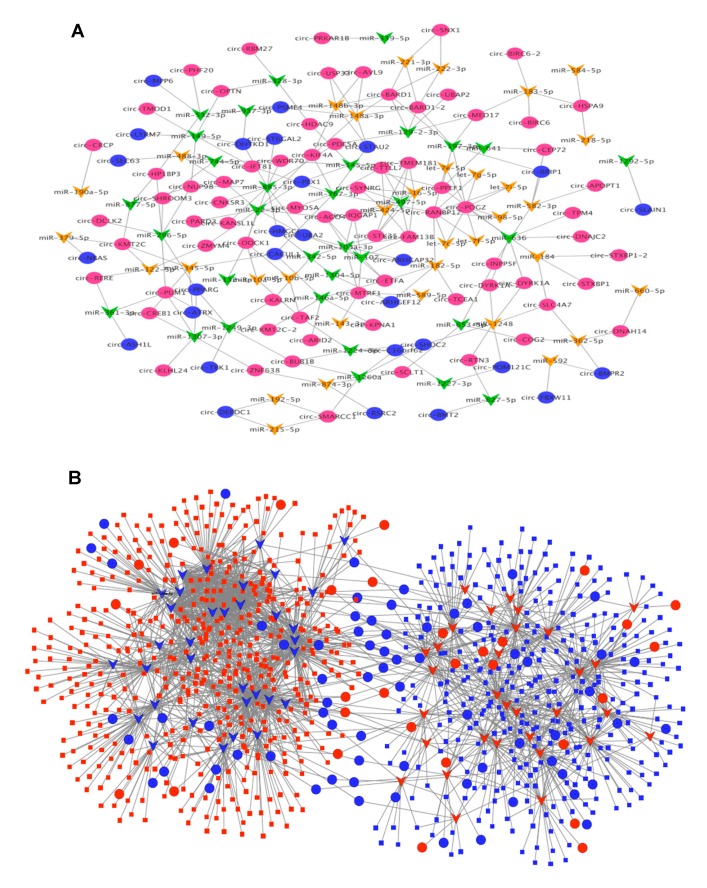
The circRNA regulatory network in neuronal activation. (**A**) The circRNA–miRNA interaction network that consists of 99 circRNAs and 65 miRNAs. The circle represents circRNA and the inverted triangle represents miRNA. The colors red and goldenrod indicate upregulation, and blue and green indicate downregulation. (**B**) The circRNA–miRNA–mRNA interaction network that contains 99 circRNAs, 65 miRNAs, and 802 mRNAs. The circle represents circRNA, the inverted triangle represents miRNA, and the rectangle represents mRNA. The red color denotes upregulation and blue denotes downregulation.

To glean further insight into the DE circRNA molecular mechanisms and the involved regulatory pathways in neuronal depolarization, we constructed the circRNAs–miRNA–mRNA association network mediated by common miRNAs that bind to the DE circRNAs and DE mRNAs. We used TargetScan algorithm with stringent context score threshold (<−0.2) settings to predict high-confidence binding sites. A total of 237 and 13,281 interactions were detected for circRNA–miRNA and circRNA–miRNA–mRNA respectively, in which 99 circRNAs, 65 miRNAs, and 802 mRNAs were associated. The data for each circRNA and its potential binding miRNAs and mRNA targets are provided in Table S4, and an entire network of circRNA–miRNA and circRNA–miRNA–mRNA interactions is illustrated in Figure 4A and Figure 4B. One significant example of the interaction that was previously established is the CDR1as/miR-7 axis [14]. We found miR-7-5p to be the third most significant miRNA, which was enhanced in depolarized cells, along with CDR1as that displayed a significant upregulation trend in this study. Also, miR-7 gene targets such as PAX6, TET2, and XIAP were found to be significantly decreased. This suggests that the CDR1as/miR-7/target genes axis is a critical pathway in neuronal activation. Some other examples of interactions of the differentially expressed RNAs are circ-PUM1/miR-145-5p/SOX9, circ-PPEF1/miR197-3p/PDGFC, and circ-CACUL1/miR-146a-5p/APPL1 (Table S4).

Considering that sponging of miRNA may not necessarily result in a detectable change in miRNA levels, we also conducted the analysis with only DE circRNA and DE mRNA using predicted miRNA mediators expressed in the cells and found that most of the modulated genes (84%) were potentially targeted by the circRNAs through binding to 324 miRNAs.

### 3.5. Subnetwork Analysis and Functional Regulatory Modules

**Figure 5:**
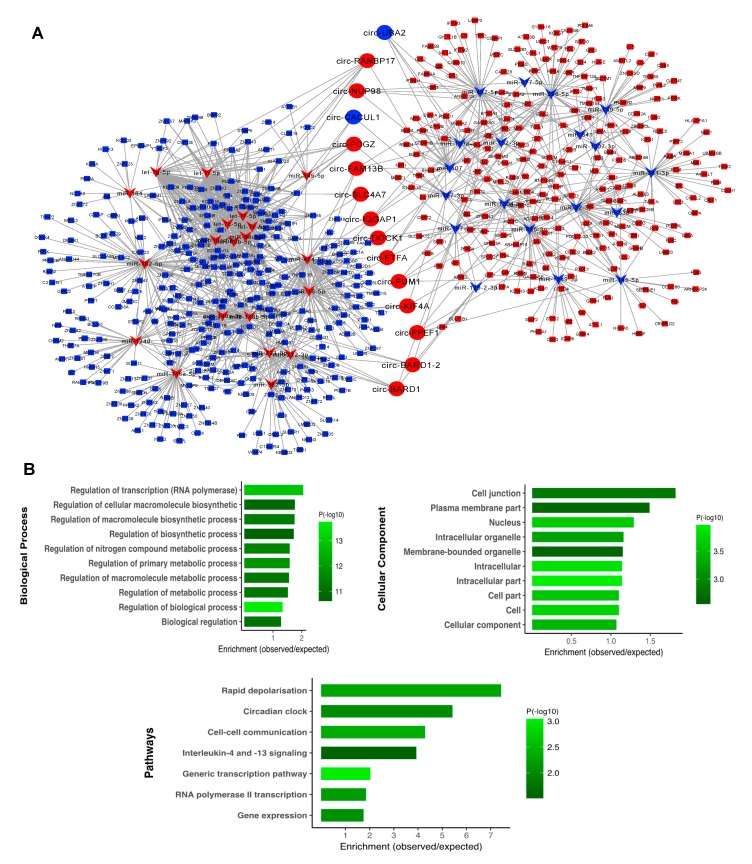
The subnetwork analysis and functional enrichment. (**A**) The top 15 highly ranked circRNA nodes from cytoHubba were used to construct the subnetwork consisting of 74 circRNA–miRNA and 1357 miRNA–mRNA pairs with 39 miRNAs and 572 mRNAs associated. The circle represents circRNA, the inverted triangle represents miRNA, and the rectangle represents mRNA. The red color denotes upregulation and blue denotes downregulation. (**B**) Functional enrichment analysis of the interacting mRNAs. The top significantly enriched GO terms and pathways are shown.

To identify the central regulatory elements in the neuronal activation process, we ranked the network nodes using 11 topological analysis methods consisting of both local- and global-based algorithms from cytoHubba plugin of Cytoscape. We found 15 high ranking circRNAs with high level overlap across all the analysis methods, for which the regulatory subnetwork was constructed. As shown in Figure 5A, this was composed of 74 circRNA–miRNA and 1357 miRNA–mRNA pairs, in which 39 DE miRNA bound to 15 DE circRNAs and DE 572 mRNA. Three circRNAs including circ-RANBP17, circ-POGZ, and circ-BARD1–2 were found to be top regulatory hubs, suggesting substantial roles for these circRNAs in the biology of neuronal excitation. To identify the associated biological processes and pathways, we performed the GO and pathways analysis for the subnetwork genes. As shown in Figure 5B, the mRNA targets were significantly enriched with several highly relevant biological processes, including transcription and metabolic process, plasma membrane, and cell junction. We also found a significant enrichment of the genes in pathways, such as early rapid depolarization and cell–cell communication.

### 3.6. Circular and Linear Transcripts Expression are Associated in Neuronal Biology

**Figure 6:**
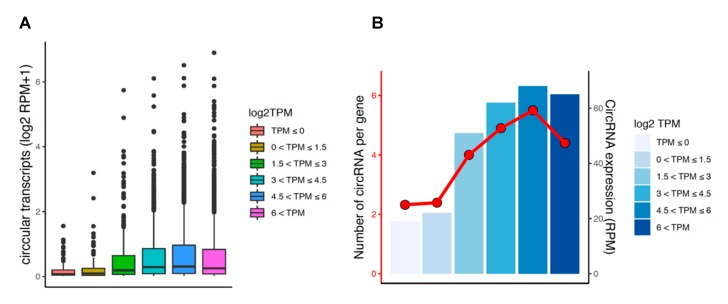
Relationship between the expression of circRNAs and their linear isoforms. (**A**) Comparison of the expression level of circular transcripts (RPM) with the linear transcripts (TPM) categorized by expression level. (**B**) Comparison of the expression level of host genes with the expression and the number of circRNAs per gene.

We explored potential association between the expression levels of circular and linear transcripts in all samples. Circular and linear expression levels were estimated in linear depleted and mRNA sequencing libraries, respectively, which prevented cross-estimation of the transcripts. We found a significant positive relationship between the expression of circRNAs and their corresponding linear transcripts (Spearman’s rank correlation test: *p* = 5 × 10^−4^; Figure 6A), suggesting that circRNA expression is co-regulated with the level of their linear isoforms. Notably, host genes with higher expression tended to produce a higher number of circRNAs, which resulted in increased average expression of the circRNAs per gene (Figure 6B). Also, in order to test the relationship between the magnitude change of circRNA and the linear isoforms in response to depolarization, we conducted correlation analysis after filtering for highly expressed circular (RPM > 0.2) and linear transcripts (TPM > 0.2) accounting for 2707 genes. The findings interestingly showed a positive relationship between changes in these transcripts (Spearman’s rank correlation test: *p* = 0.001; Figure S3). This suggests changes in circRNAs expression are associated with corresponding changes in their linear counterparts.

### 3.7. CircRNA Loss-of-Function Regulates Specific Gene Expression

**Figure 7:**
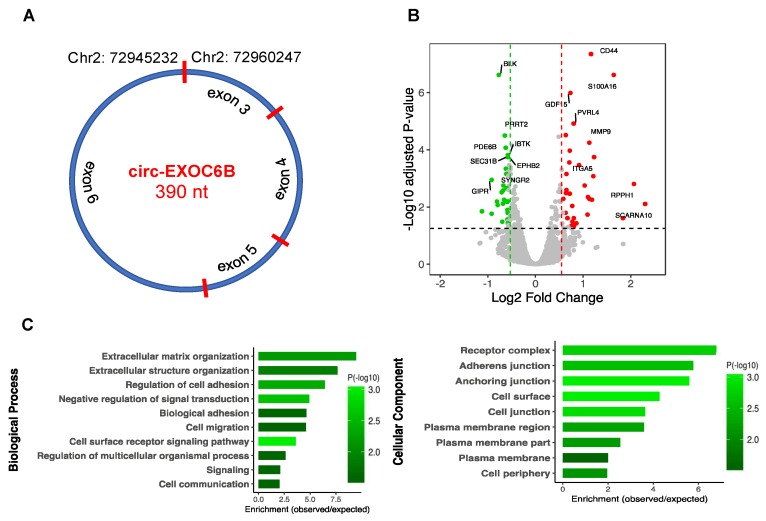
CircRNA circ-EXOC6B loss-of-function analysis. (**A**) The genomic features of circ-EXOC6B. (**B**) Volcano plot constructed using fold change values and adjusted *p*-values (FDR) to compare the gene expression changes between the control and the knockdown conditions. Red dots represent significantly upregulated genes and green dots represent downregulated genes, with the vertical lines corresponding to 1.5-fold change and the horizontal line representing an FDR cut off < 0.05. (**C**) Functional enrichment analysis of the DE genes. The top significantly enriched GO terms are shown.

To further explore the molecular function of circRNAs, we performed functional analysis on circ-EXOC6B and circ-DROSHA by siRNA knockdown in differentiated SH-SY5Y cell culture. The criteria for selecting these two circRNAs are explained as follows. Circ-EXOC6B, spanning three exons (Figure 7A), was among the top five most abundant circRNAs (average RPM > 48) with a stable expression across all the studied samples as well as in the human brain [10]. Furthermore, the host gene has been implicated in brain disorders [46]. Also, circ-DROSHA back-spliced from exons 27–29 (NM_001100412) was found the most abundant circRNA within genes involved with miRNA biogenesis and thus may affect transcriptome by modulating miRNA processing. SH-SY5Y cells were electroporated in the presence of circ-EXOC6B and circ-DROSHA siRNAs, respectively, before transcriptome analysis using mRNA-sequencing. While the lowly expressed circ-DROSHA knock down did not appear to modulate host gene nor any other gene (FDR < 0.05) (Figure S4), circ-EXOC6B was shown to significantly alter the expression levels of 60 genes (FDR < 0.05 and FC > 1.5), as shown in Figure 7B (Table S5). There were 27 genes upregulated and 33 downregulated. We crossed these to the depolarization associated genes and found 14 genes were also differentially expressed in depolarization. To explore the potential function of the circ-EXOC6B, we predicted its interaction with miRNA that potentially targets the DE genes and identified ten genes that are candidate targets for the EXOC6B binding miRNA. To interpret the biological significance of the differentially expressed genes, we performed GO annotation. Interestingly, we observed significant enrichment of terms related to the host gene function, including extracellular matrix organization, cell surface receptor signaling pathway, regulation of cell adhesion, cell migration, plasma membrane region, and plasma membrane part (Figure 7C). Pathway enrichment analysis also indicated two categories including extracellular matrix organization and intergenic surface interactions. These observations suggest that at least some circRNAs act to modulate biological processes associated with their host genes.

### 3.8. Detection of circRNAs with Protein-Coding Potential

**Figure 8:**
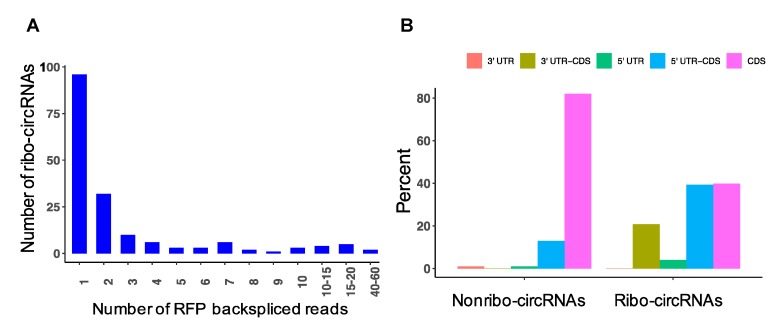
Analysis of ribosome-associated circRNAs. (**A**) The number of ribosome-associated circRNAs (ribo-circRNAs) and their back-spliced reads detected in ribosome profiling. (**B**) Annotation of the ribo-circRNAs and their comparison with nonribo (non-translated) circRNAs from RNAseq.

To investigate whether circRNAs have the potential for translation in the depolarized cells, we sequenced ribosome protected RNA fragments (RPF) by Ribo-Seq and quantified the back-splice junction spanning reads. Interestingly, we found 173 unique circRNAs, suggesting there was some evidence of active circRNA translation (Figure 8A; Table S7). The majority of the RPF back-spliced reads originated from circRNAs with medium or low abundance, not highly expressed circRNAs, indicating that the detected circRNA reads were not due to random binding. In order to get insight into the genomic composition of the ribosome-associated circRNAs (ribo-circRNAs), we performed annotation and interestingly found a significant difference compared to non-translated circRNAs from RNAseq (*p* = 1 × 10^−15^); 43% of ribo-circRNAs were spliced from the 5′-UTR region of the host genes, whereas the non-translated circRNAs were mainly derived from CDS, with only 14% spliced from the 5′-UTR region of the host genes (Figure 8B). Next, we examined whether ribo-circRNAs could potentially encode proteins by predicting highly reliable coding sequence within the exon regions of the circRNAs. The results showed that 47 ribo-circRNAs had potential to be translated into a protein (Table S7), the majority of which were in frame and partially overlapped with the host gene protein. Approximately 62% of these putative circRNAs derived proteins had at least one conserved domain, including circ-FOXO3, circ-ARNT2, circ-PTPRG, and several others, whose genes have been implicated in synaptic function (Table S7), suggesting they could support some neural function. Interestingly, nine ribo-circRNAs were derived from the genes whose circRNAs were also differentially expressed after depolarization. We compared the ribo-circRNA profiles (normalized by the total mapped reads) with depolarized and resting samples and found there were more circRNA reads in depolarized cells (*p* = 7 × 10^−10^). The top three circRNAs displaying active translation included circ-MTOR, circ-MRPL38, and circ-KSR2, which were relatively enriched in ribose reads observed in depolarized cells.

## 4. Discussion

In this study, we investigated genome-wide circRNA expression in differentiated human neuroblasts and found a remarkable diversity of species with more than 28,000 distinct molecules, more than half of which had not previously been reported in the circBase [44]. Notably, there was a significant positive correlation between the number of circRNA spliced per gene and the level of circRNA expression. Our differential analysis of these molecules after neuronal depolarization suggested there is significant alteration of circRNA expression in depolarized cells compared to their resting state and perhaps a role for circRNA in activity-associated intracellular signaling processes. These observations build on our previous investigation of miRNA–mRNA interaction in differentiated human neuroblast cells, where significant changes were observed after single and multiple depolarizing stimuli [47].

Recent evidence suggests that circRNAs can modulate transcription and fine tune miRNA function through sponging [5]. Therefore, to understand the mechanism of circRNA action in the depolarization process, we profiled the expression of miRNA and mRNA in our samples alongside circRNAand found these molecules were substantially regulated in response to neuronal depolarization. Analysis of the circRNAs–miRNA–mRNA association network revealed the potential for a large number of interactions between the differentially expressed RNAs. Prediction of the central regulatory elements suggested that 15 circRNAs were potential hubs in the network. Three circRNAs including circ-RANBP17, circ-POGZ, and circ-BARD1 were found to be top regulatory hubs, suggesting substantial roles for these circRNAs in the intracellular signaling associated with neuronal excitation. In addition to these circRNAs, we found CDR1as to be a potential regulator, as functional studies have robustly reported this circRNA to bind miR-7-5, through which many target genes are regulated [14,45]. CDR1as-deficient brains showed downregulated miR-7, suggesting an autoregulatory mechanism. This resulted in a dysfunction of synaptic transmission and behavior of mice model by dysregulating miR-7 target genes [14]. Here, we found miR-7-5p to be the third most significant miRNA with increased expression along with CDR1as, which showed a significant upregulation trend in depolarized cells. Also, miR-7 validated gene targets such as PAX6, TET2, and XIAP in addition to many predicted targets, such as ESRRG, RSBN1, and STOX2, were significantly decreased. This finding suggests that the CDR1as/miR-7/target gene is a key regulatory axis in neuronal depolarization. Activity-associated changes in the CDR1as network observed in our study are now well established in the literature [11,12,13] and provide a useful reference or positive control to support the existence of novel circRNA-based ceRNA networks that were also apparent in our data.

To further explore the functional significance of one of these molecules, we profiled the molecular consequences after siRNA knockdown. Circ-EXOC6B loss-of-function resulted in a significant change in the expression levels of many genes. Bioinformatics analysis showed this expression change could be partly mediated by miRNAs targeted by the circ-EXOC6B, and supported a causal role for circRNA in the regulation of this network. Gene enrichment analysis showed these genes are responsible for cellular processes related to the function of the EXOC68 gene and its family. This gene encodes a homolog in *Saccharomyces cerevisiae* known as SEC15, which serves as a component of the exocyst, an octameric protein complex involved in directing exocytic vesicles to specific sites on the plasma membrane and mediating tethering to the membrane prior to fusion. It is involved in numerous cell processes, including cell migration, cell growth, morphogenesis, cytokinesis, cell polarity, and primary ciliogenesis [48]. SEC15 is essential for localization of specific cell adhesion and signaling molecules required for establishment of synaptic specificity in *Drosophila* [49]. This gene promotes notch signaling and is associated with proper neuronal fate specification by mediating specific vesicle trafficking [50]. Disruption of EXOC6B was reported in a patient with intellectual disability, epilepsy, and behavioral features resembling autism [46]. It has been shown that circRNAs with retained introns can induce expression of their target genes by RNA polymerase II to the promoter region of the target genes [16]. CircRNAs can also regulate the expression of the linear counterparts by serving as competitors for translation, leading their cognate linear isoforms to escape translation, as in the case of circ-HIPK3, circ-DMD, and circ-FMN [5]. The level of circ-MBL modulates splicing of the linear transcript in favor of the circular by forming an autoregulatory loop [18]. These evidence together with our findings suggest that the circRNA regulatory role is likely related to the cognate mRNA function.

We also provided evidence of potential translation of circRNAs by ribosome profiling. Analysis of Ribo-Seq reads identified a number of circRNAs, many of which contained strong ORF sequence, enabling them to potentially produce polypeptides of various lengths. Interestingly, we observed a strong enrichment of the 5′-UTR region in the ribo-circRNAs, which is reasonable in the view of the significant role of this region in initiation and regulation of cellular translation through various mechanisms, such as ribosome complex binding motifs, IRES regions, and interacting trans-acting factors [51]. Moreover, we found many putative ribo-circRNAs proteins had at least one conserved domain, suggesting these may generate proteins with biological functions. Comparison of the ribo-circRNA profiles between depolarized and resting samples established that there were more circRNA reads in depolarized cells, suggesting that, if these circRNA are translated, they may serve some purpose in synaptic plasticity. In support of this hypothesis, several polypeptides were predicted to be generated from circRNA templates, such as MTOR, which is a critical component for synaptic plasticity, learning, and memory [52] as well as for translation of potassium channels in dendrites and neuronal membrane potential [53]. Translation of endogenous circRNAs was reported previously; for example, Pamudurti et al. indicated that a group of circRNAs encode proteins in fly heads that contain specific protein domains [19]. They revealed that circ-Mbl encodes a protein that was detected by mass spectrometry and was found to be enriched in synaptosomes of fly heads. A novel protein derived from circ-SHPRH was also found to be abundantly expressed in human brains and dysregulated in glioblastoma [21]. The circ-SHPRH protected the host protein from degradation, thus increasing the tumor suppressive activity of the gene. Similarly, circ-ZNF609 was shown to be translated in murine and human myoblasts, and the protein was suggested to modulate myoblast proliferation in response to stress conditions [20]. Other circRNAs such as circ-FBXW7 and circ-LINC-PINT were reported to be translated in brain tumors, suggesting that additional coding circRNAs have yet to be discovered. However, the study of circRNA translation is difficult due to the limitations of circRNA identification that exclusively focus on back-spliced events, which cover only a small proportion of circRNA transcripts. This results in detecting a low number of spliced RFP reads, preventing comprehensive analysis of these molecules in translation.

In summary, we provided evidence of circRNAs association with neuronal activation by showing their differential expression in depolarization of human neuroblasts. This was combined with a global change in the abundance of miRNA and mRNA in depolarized cells. In many cases, these circRNAs were altered after depolarization in accordance with their cognate mRNAs and bioinformatically associated by miRNA, supporting their action as miRNA sponges. This kind of interaction was exemplified for circ-EXOC6B by siRNA-induced loss-of-function, which demonstrated a causal relationship between circRNA specific regulation of its gene interaction network with functional significance to neural activity. Finally, we found evidence that suggests some circRNAs may serve as templates for active translation, particularly in depolarized neuroblasts.

## Supplementary Materials

The following are available online at https://www.mdpi.com/2073-4409/9/1/25/s1, Figure S1: Correlation between the number of circRNAs per gene and their average expression in the neuroblastoma., Figure S2: Validation of the identified circRNAs in neuroblastoma using q-PCR., Figure S3: Correlation between the change of circRNA and linear isoforms in response to depolarization, Figure S4: CircRNA circ-DROSHA loss-of-function analysis, Table S1: List of all the identified circRNAs in neuroblastoma, Table S2: List of differentially expressed circRNAs, Table S3: List of differentially expressed miRNAs and mRNA, Table S4: All the circRNA interactions with miRNA and mRNA., Table S5: List of differentially expressed genes for the EXOC6B KD experiment, Table S6: List of RT-qPCR divergent primers for circRNA validation, Table S7: List of ribo-circRNAs.
