# Supplementary material for: Depolarization-Associated CircRNA Regulate Neural Gene Expression and in Some Cases May Function as Templates for Translation
Source: Cells. 2019 Dec 20;9(1):25. doi: 10.3390/cells9010025 (PMC7017197; doi:10.3390/cells9010025)
Supplement: Supplementary file 1 [file cells-09-00025-s001.zip › cells-596908- revised 1 supplentary/cells-596908 revised 1 supplementary.pdf]

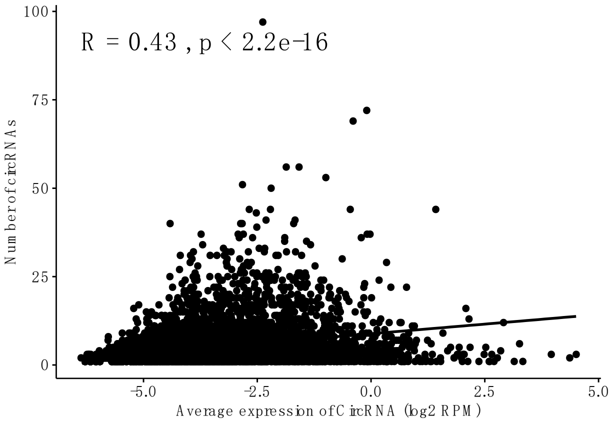

Fig. S1. **Correlation between the number of circRNAs per gene and their average expression in the neuroblastoma**. The correlation coefficient (R) and P-value were calculated with Spearman's rank correlation test. The results show some positive relationship between the number of circNRA spliced per gene and their average expression.

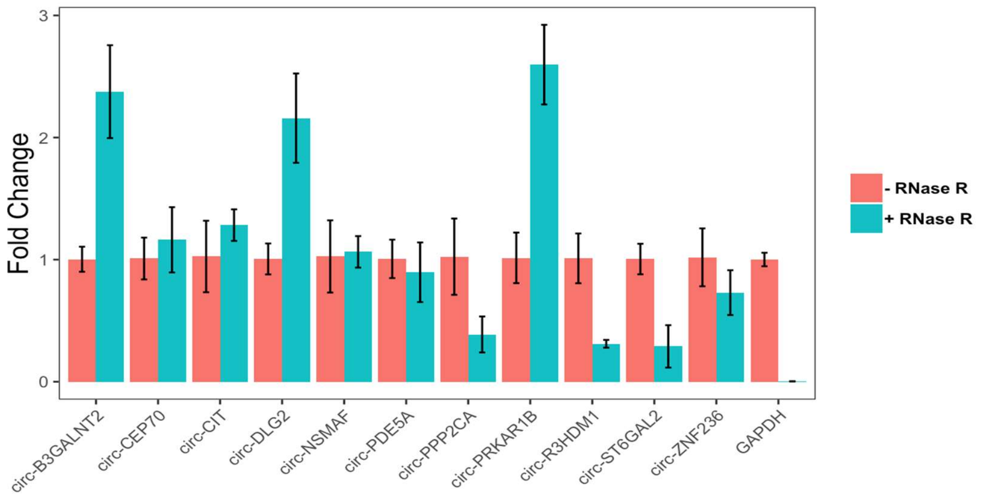

Fig. S2- Validation of the identified circRNAs in neuroblastoma using q-PCR. Ten circRNAs were tested for RNase R resistance. Data are shown as mean ± SEM. Reactions were in triplicates and circRNAs resistance was compared to GAPDH as control.

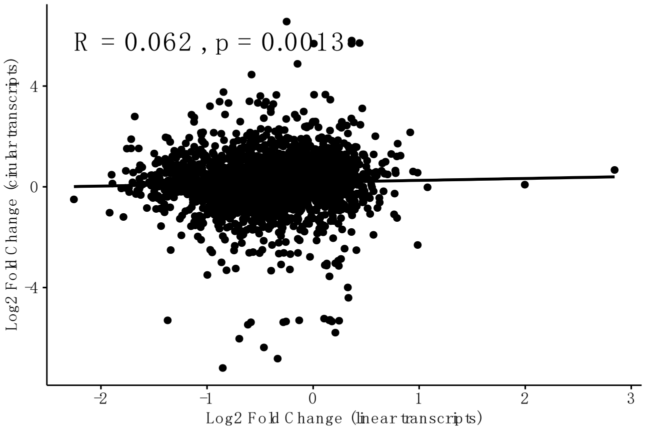

Fig. S3- Correlation between the change of circRNA and linear isoforms in response to depolarization. Only highly expressed transcripts (RPM and TPM > 0.2) were considered. The correlation coefficient (R) and P-value were calculated with Spearman's rank correlation test.

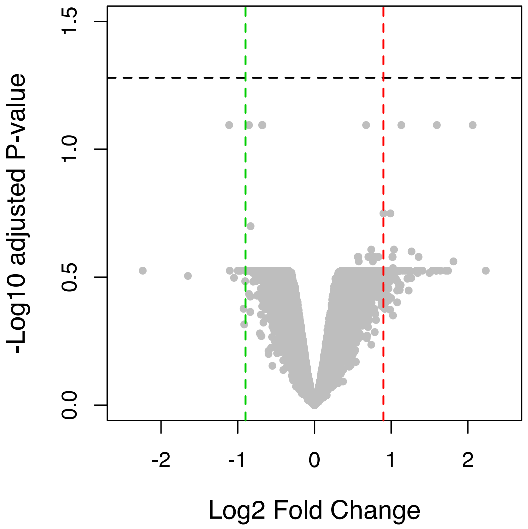

Fig. S4- CircRNA circ-DROSHA loss-of-function analysis. Volcano plot constructed using fold change values and adjusted P-values (FDR) to compare the gene expression changes between the control and knockdown conditions.The vertical lines corresponding to 1.5-fold change and the horizontal line representing a FDR cut off < 0.05.
